# Normoxic re-oxygenation ameliorates end-organ injury after cardiopulmonary bypass

**DOI:** 10.1186/s13019-020-01173-4

**Published:** 2020-06-10

**Authors:** Yun-Wen Peng, Terry Major, Azmath Mohammed, Kristopher B. Deatrick, John R. Charpie

**Affiliations:** 1 Division of Pediatric Cardiology, Department of Pediatrics, University of Michigan Medical School, 1540 East Hospital Drive, 11-740 C.S. Mott Children’s Hospital, Ann Arbor, MI 48109-4204 USA; 2 Department of Surgery, University of Michigan Medical School, Ann Arbor, MI USA

**Keywords:** Cardiopulmonary bypass, Cardioplegic arrest, Normoxia, /hyperoxia, Reperfusion, Renal injury, Brain injury

## Abstract

**Background:**

In a rabbit model of cardiopulmonary bypass (CPB) and cardioplegic arrest, we previously showed that hyperoxic myocardial reperfusion was associated with increased left ventricular (LV) systolic dysfunction and myocardial injury compared with normoxic reperfusion. The aim of this study was to evaluate in our experimental model the impact of post-CPB reperfusion conditions on other organs potentially vulnerable to ischemic injury such as the brain and kidney.

**Methods:**

After 60 min of CPB, aortic cross-clamp, and cold cardioplegic arrest, rabbits were reperfused under hyperoxic or normoxic conditions for 120 min. Left ventricular systolic contractility (LV + dP/dt) and diastolic relaxation (LV –dP/dt) were continuously recorded, and end-organ injury was assessed by measuring circulating biomarkers specific for kidney (cystatin C and creatinine) and brain injury [S100B and neuron specific enolase (NSE)]. At completion of the protocol, kidney and brain tissues were harvested for measuring oxidant stress (OS), inflammation and apoptosis.

**Results:**

Following aortic cross-clamp removal, rabbits exposed to normoxic reperfusion demonstrated preserved LV systolic and diastolic function compared with hyperoxic reperfusion (LV + dP/dt: 70 ± 14% of pre-CPB vs. 36 ± 21%, p = 0.018; LV -dP/dt: 72 ± 36% of pre-CPB vs. 33 ± 20%, p = 0.023). Similarly, CPB increased plasma creatinine, S100B and NSE that were significantly attenuated by normoxic reperfusion compared with hyperoxic reperfusion (creatinine: 4.0 ± 0.5 vs. 7.1 ± 0.8 mg/dL, p = 0.004; S100B: 4.0 ± 0.8 vs. 6.7 ± 1.0 ng/mL, p = 0.047; NSE: 57.7 ± 6.8 vs. 101.3 ± 16.1 pg/mL, p = 0.040). Furthermore, both kidney and brain tissues showed increased mRNA expression and activation of pathways for OS, inflammation, and apoptosis, that were reduced under normoxic compared with hyperoxic conditions.

**Conclusions:**

Normoxic reperfusion ameliorates cardiac, renal and neural injury compared with hyperoxic reperfusion in an in vivo animal model of CPB and cardioplegic arrest. This protective effect of normoxic reperfusion may be due to a reduction in signaling pathways for OS, inflammation, and apoptosis.

## Background

Cardiopulmonary bypass (CPB) is a form of extracorporeal support used to divert blood away from the heart and lungs during open heart surgery, and maintain circulation and oxygen delivery to end organs. In many instances, CPB is combined with aortic cross-clamping (ACC) and cardioplegic arrest to allow the surgeon to operate on a non-beating heart in a bloodless surgical field. CPB with cardioplegic arrest is associated with myocardial ischemia-reperfusion (IR) injury and a systemic inflammatory response that impacts both short- and long-term outcomes after cardiac surgery in children and adults [1–3]. Recently, in an acute experimental model of CPB with cardioplegic arrest, we showed that myocardial oxidant stress (OS), inflammation, and apoptosis were significantly increased, and associated with a concomitant decrease in ventricular systolic function [4]. We also demonstrated that after ACC removal, normoxic reperfusion via the bypass circuit was associated with an acute reduction in OS, inflammation and myocardial injury compared to the standard hyperoxic CPB currently employed for the majority of cardiac operations [4].

The aim of this study was to extend our initial experimental observations in the heart to other vulnerable organs such as the kidney and the brain that also are also at risk for injury after CPB with cardioplegic arrest. We hypothesized that in our animal model, CPB with cardioplegic arrest would lead to renal and brain injury that could be attenuated with normoxic reperfusion following ACC removal.

## Methods

### Animal model

Male New Zealand white rabbits (3.0 ± 0.2 kg) were randomly assigned to two groups: [1] normoxic reperfusion (with 21% O_2_ CPB and inspired FiO_2_ 0.21, n = 6), or [2] hyperoxic reperfusion (with 100% O_2_ CPB and inspired FiO_2_ 1.0, n = 7). The protocol was approved by the Institutional Animal Care and Use Committee at the University of Michigan. All animals received humane care in compliance with the *Guide for the Care and Use of Laboratory Animals* published by the National Institute of Health.

### Surgical preparation and experimental protocol

The surgical preparation and experimental protocol was performed as previously described [4]. Briefly, the femoral artery was cannulated for continuous arterial pressure monitoring and blood sampling. A high-fidelity 4-Fr Millar pressure catheter (Millar, Inc., Houston, TX) was placed into the LV apex for measuring LV systolic contractility (+dP/dt), and LV diastolic relaxation (−dP/dt). For veno-arterial CPB, blood was drained from the right jugular vein and infused into the right carotid artery. Following 30 min of full CPB (100 mL/kg/min), an ascending aortic cross-clamp (ACC) was placed and cold (4 °C) cardioplegic solution infused into the aortic root. The heart was surface-cooled with ice during ACC and cardioplegic arrest. After 60 min of cardiac arrest, the heart was reperfused and gradually rewarmed, and the lungs were mechanically ventilated under normoxic or hyperoxic reperfusion conditions for 60 min on partial CPB (50 mL/kg/min) followed by complete separation from bypass for an additional 60 min. Hemodynamic parameters, including mean arterial pressure (MAP), heart rate (HR), LV + dP/dt, LV -dP/dt, ECG and rectal temperature were monitored continuously using a computer equipped with a data acquisition system (PowerLab and LabChart, ADInstruments, Colorado Springs, CO).

### Biomarkers of kidney and brain injury

Blood samples were collected from the femoral artery at multiple time points (pre-CPB, and 0.5, 60, 120 min after ACC removal) for measurement of kidney injury [cystatin C (MyBioSource, San Diego, CA), and creatinine (Cayman, Ann Arbor, MI)] [5, 6] and brain injury [S100B and neuron specific enolase, NSE (ABclonal, Woburn, MA)] [7, 8].

### Tissue specimen preparation and real-time quantitative polymerase chain reaction (RTPCR)

Rabbit kidney and brain tissues were collected immediately following termination of the experimental protocol. Kidney and brain tissue from rabbits not undergoing CPB were used as reference controls. Tissues were snap frozen in liquid nitrogen and stored at − 80 °C for measurement of OS (NOX-2, NOX-4, eNOS), inflammation (COX2, IL-1β, IL-6) and apoptosis (caspase-3, BcL-2, Bax).

Total RNA from tissue was extracted using RNeasy® Fibrous Tissue Mini kit (QIAGEN) according to the manufacturer’s instructions. The cDNA product was assessed by iTaq™ Universal SYRB® Green Supermix (Bio-Rad). The PCR primer sequence for rabbit: NOX-2 (gp-91-phox) (forward: 5′-GCTTGTGGCTGTGATAAGCA-3′; reverse: 5′-ACGGCACAG CCAGTAGAAGT-3′), NOX-4 (forward: 5′-TTGGCTTTGGATTTCTGGAC-3′; reverse: 5′-TACTGGCCAGGTCTTGTTT-3′), eNOS (forward: 5′-GCATCACCAGGAAGAAGACCTT-3′; reverse: 5′-TGTGGCCTTCACTCTCTTGC-3′), COX-2 (forward: 5′-CACGCAGGTGGA GATGATCTAC-3′; reverse: 5′-ACTTCCTGGCCCACAGCAAACT-3′), IL-1β (forward: 5′-GCCGATGGTCCCAATTAGAT-3′; reverse: 5′-ACAAGACCTGCCGGAAGCT-3′), IL-6 (forward: 5′-GAAAACACCAGGGTCAGCAT-3′; reverse: 5′-CAGCCACTGGTTTTTCTG CT-3′), caspase-3 (forward: 5′-CACGGTGATGAAGGAGTC-3′; reverse: 5′-GCAAGCCTG AATAATGAA-3′), BcL-2 (forward: 5′-TGTGGCCTTTCTTTGAGTTCG-3′; reverse: 5′-CTCCCAGCCTCCGTTATCC-3′), Bax (forward: 5′-CCTTTTGCTTCAGGGTTTCA-3′; reverse: 5′-ATCCTCTGCAGCTCCATGTT-3′), GAPDH (forward: 5′-AGGTCATCCACGA CCACTTC-3′; reverse: 5′-GTGAGTTTCCCGTTCAGCTC-3′). The PCR was performed using the AB StepOnePlus™ (Applied Biosystems) under the following conditions: initial denaturation at 95 °C for 10 min, followed by 40 cycles of 95 °C for 15 s and 60 °C for 60 s. The relative amount of mRNA was measured and normalized to GAPDH mRNA. Data were calculated by the 2 −*ΔΔCt* method [4].

### Data analysis

All data are presented as mean ± S.E. Measurements of LV function and plasma biomarkers were compared over time between the hyperoxic and normoxic reperfusion groups by one-way ANOVA with Dunnett post-test, or two-way ANOVA with Sidak post-test. Differences in mRNA expression between control, normoxic, and hyperoxic reperfusion groups were assessed using Student’s t-test for unpaired comparisons. For all tests, a P value of < 0.05 was considered statistically significant. Statistical analyses were performed using GraphPad Prism 7 (GraphPad Software, Inc., San Diego, CA).

## Results

### Left ventricular function

**Fig. 1:**
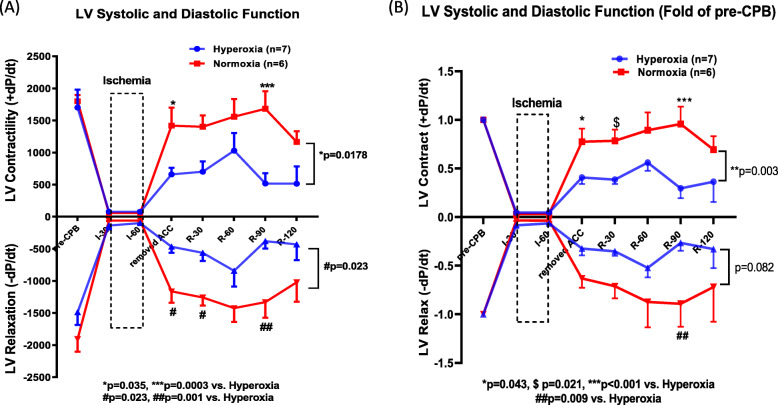
Left ventricular function. Left ventricular systolic contractility (LV + dP/dt) and diastolic relaxation (LV -dP/dt) after 60 min of global myocardial ischemia, and 120 min of normoxic reperfusion (21% O_2_ CPB and inspired FiO_2_ 0.21, n = 6; red lines), or hyperoxic reperfusion (100% O_2_ CPB and inspired FiO_2_ 1.0, n = 7; blue lines). **a** Raw data in mmHg/second, and (**b**) Fold change from pre-CPB values. All results are expressed as mean ± S.E

Baseline LV systolic and diastolic function were very similar for the normoxic and hyperoxic groups prior to ACC and cardioplegic arrest. Following 60 min of cold cardioplegic arrest and ACC removal, both LV + dP/dt and LV –dP/dt were significantly reduced in the hyperoxia rabbits (Fig. 1). For example, immediately following ACC removal, LV + dP/dt was 41 ± 7% and LV –dP/dt was 32 ± 7% of pre-CPB measurements. After 120 min of hyperoxic reperfusion (and 60 min following complete separation from CPB), LV systolic and diastolic function remained significantly impaired compared with pre-CPB values (LV + dP/dt: 36 ± 21%, p = 0.003; LV -dP/dt: 33 ± 20%, p = 0.001). In contrast, LV systolic and diastolic function were relatively preserved compared to pre-CPB measurements under normoxic reperfusion conditions (Fig. 1). For example, immediately following ACC removal, LV + dP/dt was 78 ± 14% and LV -dP/dt was 70 ± 14% of pre-CPB measurements. Similarly, after 120 min of normoxic reperfusion, LV + dP/dt was 63 ± 9% (p = 0.462), and LV -dP/dt was 72 ± 36% of pre-CPB measurements (p = 0.096). Following ACC removal, LV + dP/dt and LV – dP/dt were significantly attenuated in the hyperoxic rabbits compared with the normoxic rabbits (p = 0.018 and p = 0.023, respectively).

### Biomarkers of kidney injury

**Fig. 2:**
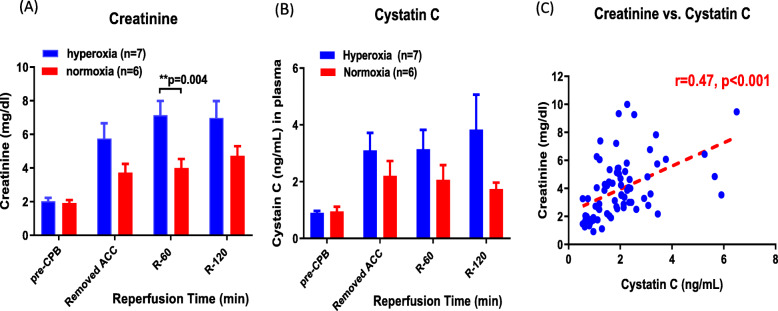
Biomarkers of kidney injury. Plasma biomarkers of acute kidney injury after normoxic (n = 6; red bars) or hyperoxic reperfusion (n = 7; blue bars). **a** Creatinine, **b** Cystatin C, and (**c**) Correlation between creatinine and cystatin C. All results are expressed as mean ± S.E

Baseline kidney function prior to CPB was similar in the hyperoxic and normoxic rabbits (Fig. 2). Immediately following ACC removal, there was a 3-fold increase in creatinine in the hyperoxic rabbits that peaked at 60-min and remained significantly elevated throughout the entire reperfusion period compared to pre-CPB levels (p = 0.0001, Fig. 2a). Rabbits exposed to normoxic reperfusion also showed a significant 2-fold increase in creatinine from pre-CPB levels (p = 0.004), but this increase in the normoxic rabbits was significantly attenuated compared with the hyperoxic group (p = 0.004).

Cystatin C was also significantly increased in the hyperoxic group compared to pre-CPB measurements (p = 0.025) with the largest increase at 120-min reperfusion (Fig. 2b). Rabbits exposed to normoxic reperfusion showed a smaller, non-significant increase in cystatin C compared to pre-CPB levels (p = 0.103). Thus, there was a trend towards higher cystatin C levels in the hyperoxic group compared with the normoxic group, but this difference was not statistically significant.

Although serum creatinine and cystatin C are reliable tests for evaluating kidney function, both may be affected by factors other than glomerular filtration rate. Figure 2c shows a moderately strong positive linear correlation between serum creatinine and cystatin C levels in our experimental model.

### Biomarkers of brain injury

**Fig. 3:**
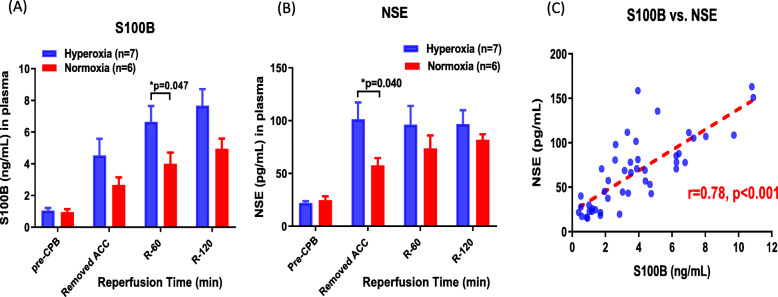
Biomarkers of brain injury. Plasma biomarkers of brain injury after normoxic (n = 6; red bars) or hyperoxic reperfusion (n = 7; blue bars). **a** S100B. **b** NSE, and **c** Correlation between S100B and NSE. All results are expressed as mean ± S.E

Baseline measures of brain injury prior to CPB were similar for both hyperoxic and normoxic groups. Immediately following ACC removal, S100B increased by 4-fold compared to pre-CPB levels in the hyperoxic rabbits, and continued to progressively increase throughout the reperfusion period (Fig. 3a). In the normoxic rabbits, there was a qualitatively similar, but attenuated and non-significant increase in S100B compared to pre-CPB levels. Thus, S100B was significantly higher in the hyperoxic group compared with the normoxic group (p = 0.047).

Comparable to S100B, there was an approximate 4-fold increase in NSE in the hyperoxic rabbits that peaked immediately after ACC removal and remained elevated throughout the reperfusion period (Fig. 3b). In contrast, NSE was increased to a lesser degree in the normoxic rabbits, and levels continued to progressively rise throughout reperfusion. Following ACC removal, NSE levels were significantly higher in the hyperoxic group compared with the normoxic group (p = 0.040).

Figure 3c demonstrates that plasma NSE and S100B levels were strongly correlated in our experimental rabbit model of CPB.

### Kidney OS, inflammation, and apoptosis

**Fig. 4:**
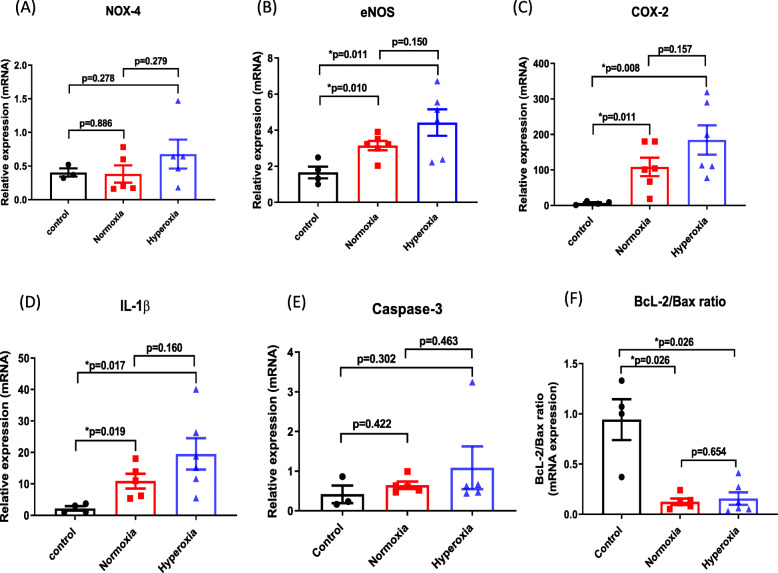
Kidney OS, inflammation and apoptosis. Relative mRNA tissue expression of markers for OS, inflammation and apoptosis in rabbit kidney. **a** NOX-4, **b** eNOS, **c** COX-2, **d** IL-1β, **e** caspase-3, and, **f** BcL-2/BAX ratio. All results are expressed as mean ± S.E. from 5 to 6 rabbits per experimental group, and 4 rabbits in the control group

In addition to measuring circulating biomarkers of kidney function, OS, inflammation and apoptosis were directly measured in renal tissue at termination of each experiment. Renal expression of the OS marker eNOS (but not NOX-4) was significantly increased in both the hyperoxic (by 4.4 ± 0.7 fold) and normoxic (by 3.6 ± 0.3 fold) groups compared to control (non-CPB) rabbits (Fig. 4a & b). Although there was a trend towards increased eNOS expression in the hyperoxic rabbits compared with the normoxic rabbits, this difference was not statistically significant.

Renal expression of the inflammatory markers COX-2 and IL-1β was also significantly increased in both the hyperoxic and normoxic groups compared to control rabbits (Fig. 4c & d). COX-2 expression was increased by 177.1 ± 44.4 fold in the hyperoxic group, and by 108.5 ± 25.9 fold in the normoxic group. Similarly, IL-1β was increased by 19.4 ± 5.0 fold in the hyperoxic group, and by 10.9 ± 2.4 fold in the normoxic group. Once again, there was a trend towards increased expression of these inflammatory markers in the hyperoxia group compared with the normoxia group, but this difference was not statistically significant.

Renal expression of caspase-3, a key enzyme that plays a central role in apoptotic signaling, was slightly increased in our experimental model of CPB with cardioplegic arrest, but this increase was not statistically significant in either cohort (Fig. 4e). In contrast, the ratio of the anti-apoptotic factor BcL-2 to the pro-apoptotic factor Bax was significantly decreased (favoring apoptosis) in both normoxic and hyperoxic conditions compared to control rabbits (Fig. 4f). There was no difference in the BcL-2/Bax ratio between normoxia and hyperoxia groups.

### Brain OS, inflammation, and apoptosis

**Fig. 5:**
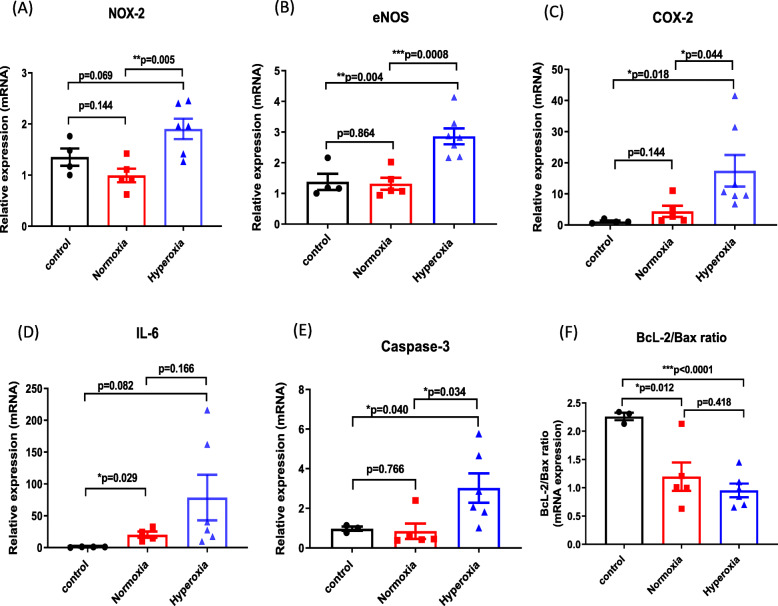
Brain OS, inflammation and apoptosis. Relative mRNA tissue expression of markers for OS, inflammation and apoptosis in rabbit brain. **a** NOX-2 (gp-91-phox), **b** eNOS, **c** COX-2, **d** IL-6, **e** caspase-3, and, **f** BcL-2/BAX ratio. All results are expressed as mean ± S.E. from 5 to 6 rabbits per experimental group, and 4 rabbits in the control group

Compared with control rabbits, brain tissue expression of the OS markers NOX-2 and eNOS were noticeably increased after hyperoxic reperfusion in our experimental model of CPB and cardioplegic arrest (Fig. 5a & b). In contrast, under normoxic reperfusion conditions, there was no increase in NOX-2 or eNOS expression compared with control rabbits. Similarly, there was increased expression of the inflammatory markers COX-2 (p = 0.018 vs. control) and IL-6 (p = 0.082 vs. control) in the hyperoxic rabbits, that was attenuated to a large degree by normoxic reperfusion (Fig. 5c & d).

Caspase-3 expression was significantly increased and the BcL-2/Bax ratio significantly decreased in brain tissue from hyperoxic rabbits compared to control rabbits (Fig. 5e & f). Furthermore, normoxic reperfusion significantly attenuated the increase in caspase-3 expression observed under hypoxic reperfusion conditions (Fig. 5e). The BcL-2/Bax ratio was not different between normoxia and hyperoxia groups (Fig. 5f).

## Discussion

Early postoperative myocardial dysfunction has a direct impact on morbidity and mortality after open heart surgery in both adults and children. Furthermore, CPB-induced acute kidney injury and brain injury have similar important (and independent) negative implications for both short- and long-term outcomes [9–12]. The various etiologies for heart, kidney and brain injury after cardiac surgery are likely variable and multifactorial, but both OS and inflammation clearly play pivotal mechanistic roles [13–15]. Perioperative strategies aimed at reducing CPB-induced OS and/or inflammation have met with limited success, and thus the majority of these therapeutic approaches have not been widely adopted into clinical practice. The observed variable responses to different therapies during open heart surgery are, at least in part, the result of individual patient-specific differences including diagnosis and procedure, genetic background, and, possibly, age- and gender-related susceptibility to OS and inflammation [16, 17].

In an in vivo experimental rabbit model of CPB with cardioplegic arrest, we previously showed that early ventricular systolic dysfunction is associated with a significant increase in myocardial OS, inflammation and apoptosis. Furthermore, normoxic versus hyperoxic reperfusion of previously ischemic myocardium attenuated the observed increase in OS, inflammation and apoptosis, and helped preserve ventricular systolic function [4]. In this study, we extended our initial observations on LV systolic contractility to include previously unpublished data on ventricular diastolic dysfunction as well. However, the primary research aim was to evaluate the impact of hyperoxic versus normoxic reperfusion strategies on kidney and brain injury in our experimental model of CPB with cardioplegic arrest. We hypothesized that similar to our observations in the heart, normoxic reperfusion would attenuate kidney and brain injury compared with hyperoxic reperfusion.

Our results showed that after CPB with cardioplegic arrest, circulating biomarkers of kidney and brain injury were significantly increased compared to pre-CPB measurements. These plasma markers of kidney [18, 19] and brain injury [20, 21] were associated with increased mRNA expression of key pathways for OS, inflammation and apoptosis in both kidney and brain tissue. Furthermore, normoxic reperfusion attenuated the increase in kidney and brain injury compared with hyperoxic reperfusion, with an overall reduction in mRNA expression of markers for OS, inflammation, and apoptosis in both kidney and brain tissue compared to hyperoxic rabbits.

In summary, these results (combined with our earlier published data) suggest that in an in vivo experimental model of CPB with cardioplegic arrest, OS, inflammation, and apoptosis are associated with acute myocardial, renal, and neurological injury and dysfunction. Furthermore, re-oxygenation under normoxic conditions attenuates myocardial and end-organ injury after IR compared with standard hyperoxic reperfusion via the CPB circuit. These experimental data support our hypothesis that CPB with cardioplegic arrest leads to myocardial, renal and brain injury that is ameliorated by normoxic reperfusion following ACC removal. The mechanism for preservation of end organ function under normoxic (compared to hyperoxic) conditions appears to be related to a reduction in OS, inflammation, and apoptosis.

There are several limitations to our experimental model and methodology that warrant further consideration and may limit the generalizability of our results. First, adult rabbits were chosen for our CPB model based on the practical considerations of animal size and cost. We recognize that there are likely age- and species-related differences in response to CPB and cardioplegic arrest, and we have recently embarked on similar experiments in a piglet model, in part, to address this question. Second, the conditions for CPB, selection of cardioplegia, hemodilution, and use of vasoactive-inotropic agents post-CPB, are all potentially important variables that could impact our results. However, these variables were the same for both experimental groups, and we have addressed these concerns previously [4]. Third, the observed organ injury and dysfunction occurred early after bypass, and the mechanisms for later brain and/or kidney injury may be quite different. Fourth, the changes in tissue mRNA expression are presumed to be global, however we recognize that the brain and kidney are not homogeneous with regards to IR tolerance and resultant protein expression. Future experiments should target specific, vulnerable areas of the brain and kidney in order to obtain more anatomically precise and comparable samples for analysis. Lastly, these experiments do not help clarify the direct impact of CPB and cardioplegic arrest on the brain and kidney versus a secondary insult from cardiac dysfunction.

## Conclusions

**Fig. 6:**
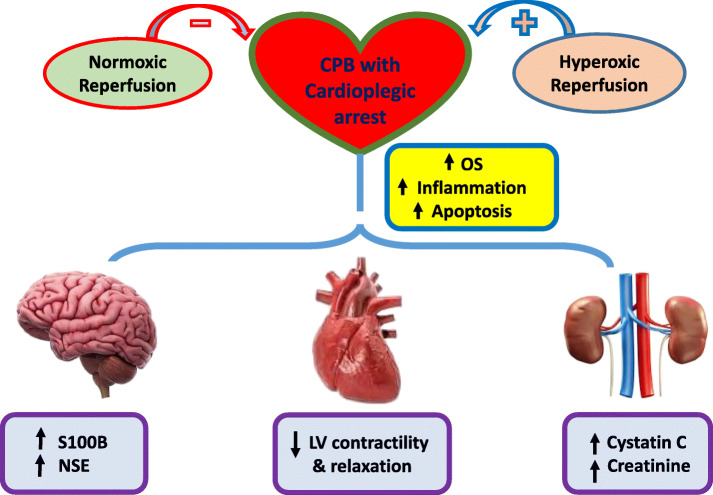
Proposed hypothesis. CPB with cardioplegic arrest is associated with an increase in OS, inflammation, and apoptosis, that contribute to acute myocardial dysfunction, kidney and brain injury. The detrimental effects of CPB and cardioplegic arrest are exacerbated by hyperoxic reperfusion conditions, and attenuated by normoxic reperfusion in vivo

Normoxic reperfusion after CPB and cardioplegic arrest ameliorates LV dysfunction, brain and kidney injury (Fig. 6). These results may have important implications for cardiac surgery programs that typically employ hyperoxic reperfusion conditions after CPB as a standard practice. At a minimum, these findings serve as preclinical data to support the design of a randomized clinical trial comparing outcomes after normoxic versus hyperoxic reperfusion in patients undergoing cardiac surgery with CPB and cardioplegic arrest. Such a targeted, preventive therapeutic approach may lessen organ injury and dysfunction, and reduce morbidity associated with CPB [22]. In addition, further understanding of the pathophysiology of organ injury after CPB may uncover new therapeutic targets to help improve multi-organ function and recovery [23, 24].

## Supplementary information

**Additional file 1 Appendix**. The RT-PCR primer sequence for rabbit

## Data Availability

The datasets used and /or analyzed during the current study are available from the corresponding author on reasonable request.

## Abbreviations

CPB: Cardiopulmonary bypass
IR: Ischemia-reperfusion
OS: Oxidant stress
ROS: Reactive oxygen species
ACC: Aortic cross-clamp
