# Supplementary material for: Normoxic re-oxygenation ameliorates end-organ injury after cardiopulmonary bypass
Source: J Cardiothorac Surg. 2020 Jun 10;15:134. doi: 10.1186/s13019-020-01173-4 (PMC7285475; doi:10.1186/s13019-020-01173-4)
Supplement: Supplementary file 1 — Additional file 1 Appendix. The RT-PCR primer sequence for rabbit [file 13019_2020_1173_MOESM1_ESM.pdf]

Appendix

The RT-PCR primer sequence for rabbit:

1) NOX-2 (gp-91-phox) (forward: 5’-GCTTGTGGCTGTGATAAGCA-3’; reverse: 5’-ACGGCACAGCCAGTAGAAGT-3’), NM_001082100.1
2) NOX-4 (forward: 5’-TTGGCTTTGGATTTCTGGAC-3’; reverse: 5’-TACTGGCCAGGTCTTGCTTT-3’), XM_002708641.3
3) eNOS (forward: 5’-GCATCACCAGGAAGAAGACCTT-3’; reverse: 5’-TGTGGCCTTCACTCTCTTTGC-3’), NM_001082733.1
4) COX-2 (forward: 5’-CACGCAGGTGGAGATGATCTAC-3’; reverse: 5’-ACTTCCTGGCCCACAGCAAACT-3’), U97696.1
5) IL-1β (forward: 5’-GCCGATGGTCCCAATTACAT-3’; reverse: 5’-ACAAGACCTGCCGGAAGCT-3’), NM_001082201.1
6) IL-6 (forward: 5’-GAAAACACCAGGGTCAGCAT-3’; reverse: 5’-CAGCCACTGGTTTTTCTGCT-3’), NM_001082064.2
7) caspase-3 (forward: 5’-CACGGTGATGAAGGAGTC-3’; reverse: 5’-GCAAGCCTG AATAATGAA-3’), NM_001082117.1
8) BcL-2 (forward: 5’-TGTGGCCTTCTTTGAGTTCG-3’; reverse: 5’-ATCCCAGCCTCCATTATCCT-3’), XM_008261439.2
9) Bax (forward: 5’-CCTTTTGCTTCAGGGTTTCA-3’; reverse: 5’-ATCCTCTGTAGCTCCATGTT-3’), XM_008252361.2
10) GAPDH (forward: 5’-AGGTCATCCACGACCACTTC-3’; reverse: 5’-GTGAGTTTCCCGTTCAGCTC-3’). NM_001082253.1

Oryctolagus cuniculus cytochrome b-245 beta polypeptide (CYBB), mRNA

NCBI Reference Sequence: NM_001082100.1

GenBank Graphics

```
>NM_001082100.1 Oryctolagus cuniculus cytochrome b-245 beta polypeptide
(CYBB), mRNA
AAGCAGCACAGTATAGAAGTGCAGACACAACACGGTGCACCTCTGCCACCATGGGGAACTGGGTTGAGAA
TGAAGGACTCTCCATCTTCGTTATTTTCGTTTGGCTGGGGCTGAACGTCTTCCTCTTTGTGTGGTACTAC
TGGGTTTATGCTTTTGGAAAGAAGTATTATTATACTACAAAACTTCTTGGGGCAGCACTGCCATTGGCCA
GGGCCCCAGCAGCCTGCCTGAACTTCAACTGCCTGCTGATCCTGCTTCCAGTCTGTCGAAATCTGCTCTC
TTTTCTCAGGGGTTCCAGTGCGTGCTGCTCAACAAGAATTCGAAGACAACTGGACAGAAATCTCACTTTT
CATAAAATGGTGGCGTGGATGATTGCACTTCATTCTGCGATTCACACAATTGCACATCTATTCAATGTGG
AGTGGTGTGTGAATGCCCGAGTCAACAATTCTGATAGTTACTCGATAGCACTCTCTGAAATTGGGGACAA
AAAGAATGAAACTTACCTCAATTTTGTTCGAGAGAGAATCAAGAACCCTGAAGGAGGCCTGTTAGTGGCT
GTAACGCGGTTGGCAGGCGTCACTGGAATTATCATCACACTGTGCCTCATACTTATCATCACATCCTCCA
CCAAAATCATCCGGAGGTCTTACTTTGAAGTGTTTTGGTACACACACCATCTCTTCGTCATATTCTTCAT
TGGTCTTGCCATCCATGGAGCTGAACGAATTGTACGTGGGCAAACTGAGGAGAGTCTGAAGAAACATGAT
CCAGTGATGTGTGAGCAACATATCTCAGATTGGGGAAAGATAAAGGACTGCCCTGTCCCAGAATTCTCTG
GAAACCCTCCTATGACCTGGAAATGGATAGTGGGTCCCATGTTCCTATATCTCTGTGAGAGGTTGGTCCG
```

GTTTTGGCGATCTCAGCAGAAGGTGGTCATCACCAAGGTGGTCACTCACCCTTTCAAAACCATTGAACTA
CAAATGAAGAAGAAAGGATTCAAGATGGAGGTGGGACAATATATTTTTGTCAAGTGCCCCACGGTGTCCA
AGCTGGAATGGCACCCTTTCACACTGACCTCTGCCCCTGAAGAAGACTTCTTTAGCATCCATATCCGCAT
CGTGGGGGACTGGACAGAGGGGCTGTTCAATGCTTGTGGCTGTGATAAGCAGGAGTTTCAAGATGCATGG
AAACTACCTAAGATAGCAGTTGATGGGCCCTTTGGCACAGCAAGTGAAGATGTGTTCAGCTATGAGGTGG
TGATGTTAGTGGGAGCAGGAATTGGGGTCACGCCCTTTGCATCCATCCTCAAGTCAGTCTGGTACAAATA
TTGTGACAATGCCACGAATCTGAGGCTCAAAAAGATCTACTTCTACTGGCTGTGCCGTGACACACATGCC
TTTGAGTGGTTTGCAGACTTGCTGCAGCTGCTGGAGACACAGATGCAGGAGAGGAACAATGCTGGCTTCC
TCAGCTACAACATCTATCTCACCGGCTGGGATGAGTCCCAGGCCAATCACTTTGCTGTGCACCATGATGA
GGAGAAAGATGTGATCACAGGTCTGAAACAAAAGACACTGTATGGACGACCCAACTGGGATAATGAGTTC
AAGACAATTGCAAGTCAACACCCTAATACCAGAATAGGAGTTTTCCTCTGTGGACCTGAAGCCTTGGCTG
AAACCCTCAGTAAACAGAGCATCTCCAACTCTGAGTCTGGCCCTCGGGGAGTGCACTTCATTTTCAACAA
GGAAAACTTCTGATTCATCCCACTACGAGGAAAGAAATTTGGGTTATGCTGCAAAGTGCCCAAATAACTC
TGGTTAATAATATAAGATCCTCTCTCTTTAAAAAAAA

PREDICTED: Oryctolagus cuniculus NADPH oxidase 4 (NOX4), mRNA

NCBI Reference Sequence: XM_002708641.3

GenBank Graphics

>XM_002708641.3 PREDICTED: Oryctolagus cuniculus NADPH oxidase 4 (NOX4), mRNA
CGGGAATCAATCAGTCCATAAGAAGTAGTTGTGTGCGTGACGAACTGAGGGGTGGGGTGGGGCGGGGGTC
CCATTCTCAGCAGCTCTGCGTGTCCGGCTGCACACCTCTGCGGGTGGTGTGGAGCAGGGCAGGCCGGGCT
AGACTGCGGGGACGCCTCCGAGTTCCTTACCCGGGACATCCTGAGCGGCGGCAGCCACAACAACGGGCTC
GCCCCCAGACAAAGGGCCGGCGCGGCGGCGAACACTGCACAGCCGGGCAGCAGCCGCGGGGCGCGCTGCG
GGCGCCAGGTCGGGCCGCAGACCGGCCAGGGCAGACGCGGCGCGGGTCGAGCCGGGTCGCGGAGCCCGCC
CGGGCTGGCTCGGTGCCGCGCCTCGGAACTTCCACTCAGTCTTTGACCCCGGGTCCCCCGCCGCCCCCGC
CCCCGCAGGCCGCGGCACTGTAACCGCTGCCTGCGCCGCCGCGCGCTCCTTTTCGGTGTGCAGGCGTGGA
GCGCAGCACCGGGGCCGACGGCATGGATGTGTCCTGGAGAAGCTGGCTGGCCAACGAAGGGGTTAAACAC
CTCTGCCTGCTCATCTGGCTTTCCCTGAATGGTCTGCTTTTCTGGAAAACCTTCCTGCTGTATAACCAAG
GGCCAGAGTACCATTACCTCCACCAGATGTTAGGCCTAGGATTGTGTCTAAGCAGAGCCTCCGCATCTGT
TCTTAACCTCAATTGCAGCCTCATCCTTTTACCCATGTGCCGAACACTCCTGGCTTACCTTCGGGGATCC
CAGAAGGTCCCAAGCAGGAGAACAAGAAGAGTGTTGGATAAGAGCAGAACATTCCATATAACTTGTGGTG
TCACTATCTGTATTTTCTCAGGTGTGCATGTGGCTGCCCATCTGGTGAATGCCCTCAACTTCTCAGTGAA
CTACAATGAGGATTTTATTGAACTGAATGCAGCAAGATACCGAGATGAGGATCCTAGAAAGCTTCTCTTC
ACAACTGTTCCTGGCCTGACAGGAGTCTGCATGGTAATGGTGCTATTCCTCATGGTTACAGCTTCTACTT
ATGCAATAAGAGTTTCTAACTATGACATCTTCTGGTATACTCATAACCTCTTCTTTGTCTTCTACATGCT
GCTGATGTTGCATGTGTCAGGGGGGCTGCTGAAGTATCAAACTAATTTAGATACCCACCCTCCTGGCTGC
ATCAGTCTTAACCAAACTCACCATCAAGACATTTCCTTGCCATTGCATCACTCAGAACATTTGCCTGGAT
CTTTCTTGGGAGAATTTTCAAAACCAGAAGAACTTGCTCAGAACACATTCATGAAGATTTGTGTGGAAGA
GCCCAGATTCCGAGCTACTTTTCCACAGACTTGGCTTTGGATTTCTGGACCTTTGTGCCTATACTGTGCC
GAAAGACTTTATAGGTGTATTCGGAGCAATAAACCAGTCACCATCCTCTCAGTTATAAGTCATCCCTCAG
ATGTCATGGAAATCCGAATGGTCAAAGAAAATTTTAAAGCAAGACCTGGCCAGTATGTTATTCTGCATTG

TCCCAGTGTGTCTGCATTAGAAAACCATCCCTTTACCCTCACAATGTGTCCTACAGAAAAAAAAGCAACA
TTTGGAGTCCATCTTAAAATAGTAGGAGACTGGACAGAACGATTCCGAGATTTACTGCTGCCTCCATCTA
GTCAAGACTCTGAGATTCTGCCGTTCATTCAAACTAGAAATTACCCAAAGCTATACATAGATGGTCCATT
TGGAAGTCCATTTGAGGAGTCACTGAACTATGAGGTCAGCCTGTGCGTGGCTGGAGGCATTGGAGTGACT
CCTTTTGCATCGATACTCAACACCCTGCTGGATGACTGGAAGCCGTACAAACTTAGAAGACTGTATTTTA
TTTGGGTGTGCAGAGACATCCAATCCTTCTGTTGGTTTGCAGACTTACTCTGCATGTTATATAACAAGTT
TTGGCAAGAGAACAGACCTGACTATGTCAACATCCAGCTGTACCTCAGTCAAACAGATGGGATTCAGAAG
ATAATTGGAGAAAAATACCATGCATTGAATTCAAGACTTTTTATTGGGCGTCCTCAGTGGAAACTTCTAT
TTGATGAAATAGCAAAATATAACAGAGGGAAAACAGTTGGTGTCTTCTGCTGCGGACCCAATTCACTTTC
CAAGATTCTTCATAAACTGAGTAACCAAAGCAACTCATATGGGACAAGATTTGAATACAATAAAGAATCT
TTCAGCTGAAAATCTGAGAAGAACCAGGACTCTAAAGAAGGAATGTGTGCAATATCTAAAACTTTGAAAC
TCAGCTGAATCAAACAGCTTTGCTGTGCCAAAGAGTAGCAAGGTTTTATTATTTATGATTATTTAAAATG
GAAATGACAGAGAATGTGGCAAGATGACAAGCCACATGCATGTTTGATCTGGAAACCAGAGAGGCCCTGA
GGATATTTGATGTGATGATTCACTTTTCATTGCTCAAATTAAAAGAAAACTATTAGATGCTCACTGTTGA
TTCTTATAGCAGTGTCAAGAACTCCCTAGTGAGGAGCTGAACTCCGTCACTCTGAGGCTGACAGTCATGG
TCCTCTTTAAATTGTTTATGGTTGAACAAATGAAAGAAGAAAATTAAAAATTCATTGAAACTCAGATTAT
GTTTTCTACATTGTGTATAAACACAATAGCTTTAATTTGGAAAATTTCCAGGCATATATATTAGATGTCT
TAAAATACAGCACGAGACTCTGTATTGATACGGGTACTTTGTGTTTAATCTTCCTCACTACTGATAATAC
CTCTGCTTGTTGTCTGTAGGCTATATGTAGTATCTACTTTTATATTTTTCTCTCTTCCTTTTATTCTAAC
AAGGAAAGTCTCTCCTTTCCTAGCCTACATTAGACTCAATAATGTAATAGTTCATTTTCATCTTCAACTT
ATTTAATATATGATTATTGCATAATATGAAAATATAATTACTATAATTGTAAAGGGACATGTATCCTAGT
AACATTTTTTCTTTAGCATTGTGGTCTCTATTTTTTTTTCCACTTTTATTCCTAGGCACACTTTATCTTT
TATTATTTTATTTTATTTTGCCTTTTAAAGTGCATTCTTTAATTTGTGCTTTGTGACTGAAGGACATCAT
ACCTGCCAGCAGGCACTGAGAAACAGTATCTCTATCCCTTATCACACAATGCTTTGTGATGGAGATTTTA
AAAGTGTAGTCACATTCCTGAATATGAACCCTGTATTTTACTTTCTCCATAAAAGGAAATCCATCATCAG
TCATTCAAATTTATTTTTCAGAATCAGTGTTATTGTCTCACTGTTTGATATCCAACATTCTAAGAGAATT
GTTGGGTTTAAAGTTGCTGTAGGGACTACACTGTGTGACCCTATCAGTTTGTTTCCTGACTTTTATATTT
GCTACCTTAATAATTAAGGAGTTCATATAGCTTAAAGATTTTGTTTAGTGGATATTTATCAAATGCTATG
AAATATAACACACTCTGATTTAAGCAGTTTGATTTGCCTCTGTTAAAATTTTTGCATACAACATAACATG
ATGGATGCTACTGTCAGTTTTTTTTTTTGTTAGGATTCACCAATAGCAGTCAAGCATGCTAAACCACCCA
ACGCTGAATGACTGTGAGTATATTTACATGTACACATATCTTCCTTCCCTTCTAATTTCTTCCTTTCCCT
TCTGTAGAAAGGAGGTGAATCAGAAAAAATGATGGGAAATGTGCAATGCAACTGCAGATTGAGCTCTGAG
GGCATAGTTAATAACCAGTGCATCTTGGCTACACTTCTTAGAGTAAAAATAATTTTATGAAAAACCTGTA
ACAGAAAGAACTAATAAAAAAAGTTTGAATTTGTTCAGGCTTAATGTGCAATACTGACTCTTTAATGATA
ACTTATATATGTGATTATATTAATGCACCTAATTTCAATAAAACAGATCCTTCCATCAGTTTTTCA

Oryctolagus cuniculus nitric oxide synthase 3 (NOS3), mRNA

NCBI Reference Sequence: NM_001082733.1

GenBank Graphics

>NM_001082733.1 Oryctolagus cuniculus nitric oxide synthase 3 (NOS3), mRNA
ATGGGCAACTTGAAGAGTGTGGGCCAGGAGCCCGGGCCACCCTGTGGCCTGGGGCTGGGGCTGGGCCTCG

GGCTGTGCGGCAAGCAGGGCCCAGCCTCCCCAGCACCAGAGCCCAGCCGGGCGCCCGCATCCCCACCCCC
AACCCCTCCAGCGCCAGACCACAGCCCCAAACCTAGCTCCAACAGTCCTCCGCTAACTCGGCCCCCGGAG
GGGCCGAAGTTCCCTCGAGTGAAGAACTGGGAGGTGGGCAGCATCACCTACGACACCCTCAGTGCCCAGG
CACAGCAGGACGGGCCTTGCACCCAAAGACGCTGCCTGGGCTCCCTTGTGTTTCCCCGGAAACTGCAGAG
CCGGCCCACCCAGGGCGCTCCACCCCCTGAACAGCTGCTGGTTCAGGCCAGGGACTTCATCAACCAGTAC
TACAGCTCCATCAAGAGGAGCGGCTCCCAGGCCCATGAGCAACGGCTTCAGGAAGTGGAGGCCGAGGTGG
CAGCCACAGGCACCTACCAGCTTCGGGAAAATGAACTGGTCTTTGGGGCCAAGCAGGCCTGGCGCAACGC
TCCCCGCTGTGTGGGCCGGATCCAGTGGGGGAAGCTGCAGGTCTTCGATGCCCGGGACTGCAGGTCTGCA
CAGGAAATGTTCACCTACATCTGCAACCACATCAAGTATGCCACCAACCGGGGCAACCTTCGTTCAGCCA
TCACAGTGTTCCCCCAGCGCGGCCCGGGCCGCGGAGACTTCCGGATCTGGAACAGCCAGCTGGTGCGCTA
TGCGGGCTACAGACAGCAGGACGGCTCTGTGCGGGGGGACCCAGCCAACGTGGAGATCACCGAGCTCTGC
ATCCAGCACGGCTGGACCCCAGGAAACGGGCGCTTCGATGTGCTGCCCCTGCTGCTTCAGGTCCCGGACG
AGCCCCCAGAACTCTTCACTCTGCCCCCTGAGTTGGTCCTGGAGGTGTCCCTGGAGCACCCCACGCTGGA
GTGGTTCGCGGCCCTGGGTCTTCGCTGGTATGCCCTCCCGGCAGTGTCCAACATGCTGCTGGAAATCGGG
GGGCTAGAGTTCCCTGCAGCCCCTTTCAGCGGCTGGTACATGAGCACGGAGATCGGCACACGGAACCTGT
GTGACCCTCACCGCTACAACATCCTGGAGGATGTGGCCGTCTGCATGGACTTGGATACCCGGACAACGTC
GTCCCTGTGGAAAGACAAGGCGGCGGTGGAGATTAACTTGGCTGTGCTGCACAGTTACCAGCTCGCCAAA
GTGACCATTGTGGATCACCACGCTGCCACAGCGTCCTTCATGAAGCACCTGGAGAATGAGCAGAAGGCCA
GAGGGGGCTGCCCTGCTGACTGGGCCTGGATCGTGCCCCCCATCTCGGGCAGCCTCACTCCTGTCTTCCA
TCAAGAGATGGTCAACTATTTCCTGTCCCCTGCCTTCCGCTACCAGCCAGACCCCTGGAAGGGGAGTGGA
GCCAAGGGCACTGGCATCACCAGGAAGAAGACCTTTAAGGAAGTGGCCAATGCAGTGAAGATCTCTGCCT
CACTCATGGGCACAGTGATGGCAAAGAGAGTGAAGGCCACAATCCTGTACGGCTCCGAGACTGGCCGGGC
CCAGAGCTACGCGCAGCAGCTGGGGAGGCTCTTCCGGAAGGCGTTCGACCCCCGGGTTCTGTGCATGGAC
GAGTATGATGTGGTGTCCCTCGAACACGAGACGCTGGTGTTGGTGGTGACCAGCACATTTGGGAATGGAG
ATCCACCAGAGAACGGAGAGAGTTTTGCAGCAGCCCTGATGGAGATGTCCGGCCCCTACAACAGCTCTCC
GCGGCCAGAGCAGCACAAGAGTTACAAAATCCGCTTCAACAGCGTCTCCTGCTCAGACCCGCTGGTGTCT
TCTTGGCGGCGGAAGAGGAAGGAGTCCAGTAACACAGACAGTGCAGGGGCTCTAGGCACCCTCAGGTTCT
GTGTGTTCGGCCTGGGCTCCCGGGCATACCCCCACTTCTGTGCCTTTGCTCGTGCGGTGGACACGCGGCT
GGAAGAGCTGGGCGGGGAGCGGCTGCTGCAGCTGGGCCAGGGCGATGAGCTATGCGGCCAGGAGGAGGCC
TTCCGCGGCTGGGCCCAGGCTGCCTTCCAGGCCTCCTGTGAGACTTTCTGCGTGGGAGAGGATGCCAAGG
CTGCTGCCCGGGACATATTTAGCCCCAAACGGAGCTGGAAGCGCCAGAGGTACCGGCTGAGTACCCAGAC
CGAGGGCCTACAGTTGCTGCCAGGTCTGATCCACGTGCACAGGCGGAAGATGTTCCAGGCCACAATCCTC
TCAGTAGAAAACCTGCAAAGCAGCAAATCCACCCGGGCCACAATCCTGGTGCGTCTGGATACTGGTGGCC
AGGAAGGGCTACAGTACCAGCCAGGGGACCACATAGGAGTCTGCCCACCCAACCGGCCTGGCCTCGTGGA
GGCGCTGCTGAGCCGTGTGGAGGACCCGCCGCCACCCACGGAGCCTGTGGCAGTGGAGCAGCTGGAGAAA
GGCAGCCCTGGTGGCCCTCCCCCCAGCTGGGTGCGGGACCCCCGGCTGCCCCCGTGCTCGCTGCGCCAGG
CTCTCACCTTCTTCCTGGACATCACCTCTCCATCCAGTCCTCGACTCCTTCGACTGCTCAGCACCTTGGC
TGAAGAGCCCAGCGAACAGCAGGAGTTGGAGGCCCTCAGCCAGGACCCCCGGCGCTACGAGGAGTGGAAG
TGGTTCCGCTGCCCCACGCTGCTGGAGGTGCTGGAGCAATTCCCGTCCGTGGCACTGCCTGCTCCCCTGC
TCCTCACCCAGCTGCCCCTGCTGCAGCCCCGGTACTACTCAGTCAGCTCGGCACCCAGCATCTACCCAGG
AGAGATCCACCTGACTGTCGCTGTGCTGGCCTACAGGACCCAGGATGGGCTGGGCCCCCTGCACTATGGA

GTCTGCTCCACGTGGCTGAGCCAACTCAAGGCTGGAGACCCCGTGCCCTGCTTCATCAGGGGGGCTCCCT

CCTTCCGGCTGCCACCTGATCCCAGCTTGCCCTGCATCCTGGTGGGCCCCGGCACCGGCATCGCCCCCTT

CCGGGGGTTCTGGCAGCAGCGCCTGCATGACATTGAGAGCAAAGGGCTGCAGCCCGCGCCCCTGACTCTG

GTGTTCGGCTGCCGATGCTCCCAGCTGGATCACCTGTACCGCGACGAGGTGCAGGACGCCCAGCAGCGCG

GAGTGTTTGGCTGCGTCCTCACCGCCTTCTCCAGGGAACCCGACAGCCCCAAGACCTACGTGCAGGACAT

CCTGAGGACCGAGCTGGCGTCTGAGGTGCACCGCGTGCTGTGCCTCGAACGCGGCCACATGTATGTCTGC

GGAGATGTCACCATGGCAACCAGCGTCCTGCAGACGGTGCAGCGAATCCTGGCCACCGAGGGCGACATGG

AGCTGGACGAGGCCGGTGACGTCATCGGCGTGCTGCGGGATCAGCAACGCTACCACGAGGACATTTTCGG

GCTCACGCTGCGCACCCAGGAGGTGACAAGCCGCATACGCACCCAGAGTTTCTCCTTGCAGGAGCGGCAG

CTGCGGGGCGCGGTGCCCTGGGCGTTCGACCTACCCGGCTCCGACACCCCCGGCCCCTGA

Oryctolagus cuniculus cyclooxygenase-2 (COX-2) mRNA, complete cds

GenBank: U97696.1

GenBank Graphics

>U97696.1 Oryctolagus cuniculus cyclooxygenase-2 (COX-2) mRNA, complete cds

GCACGAGTGCACGCCCAGCTCCGCCCGCCGCCCCGGACGCCCGCGCCCGCCGAGATGCTCGCCCGCGCCC

TGCTGCTCTGCGCCGCGGTGGCGCTCAGCCACGCAGCAAATCCTTGCTGTTCCAACCCATGTCAAAACCG

AGGTGTGTGCATGACCATGGGATTTGACCAGTACAAGTGCGACTGCACCCGGACCGGATTCTACGGTGAA

AACTGCTCCACGCCGGAATTTCTGACCAGAATCAAACTGCTCCTGAAACCCACTCCGGACACGGTGCACT

ACATCCTTACGCACTTCAAGGGAGTCTGGAACATCGTCAATAGCATTCCTTTCCTGCGAAATTCAATCAT

GAAATACGTGCTGACATCCAGATCACATATGATTGACAGTCCACCAACATATAATGTGCACTACAACTAC

AAGAGCTGGGAAGCCTTCTCTAACCTCTCCTACTACACAAGAGCCCTCCCCCCTGTGGCCGATGACTGCC

CAACGCCCATGGGTGTGAAAGGCAAGAAAGAACTTCCTGACTCTAAAGACGTGGTGGAGAAACTTCTCCT

CAGAAGAAAGTTCATCCCCGACCCCCAGGGCACCAACATGATGTTTGCATTCTTTGCCCAGCACTTCACC

CACCAGTTCTTCAAGACAGATCTTAAGCGGGGACCAGCCTTCACCAAAGGACTGGGACATGGGGTGGACT

TAAATCATATTTACGGTGAAACTCTGGACAGACAGCATAAACTGCGCCTCTTCAAGGATGGAAAAATGAA

ATATCAGGTAATTGATGGAGAGGTATATCCTCCAACAGTCAAGGACACGCAGGTGGAGATGATCTACCCG

CCTCACATCCCTGCGCACCTGCAGTTTGCTGTGGGCCAGGAAGTGTTTGGTCTGGTGCCTGGTTTGATGA

TGTATGCCACAATCTGGCTCCGGGAACATAACAGAGTGTGCGATGTGCTCAAACAGGAGCATCCAGAATG

GGATGATGAGCAGCTATTCCAGACAAGCAGGCTAATACTGATAGGAGAGACTATTAAGATTGTGATTGAA

GACTATGTACAGCATTTGAGTGGCTATCACTTCAAACTCAAGTTTGACCCAGAGCTGCTTTTCAACCAAC

AATTCCAGTACCAAAACCGGATTGCTGCTGAGTTTAACACACTCTATCACTGGCATCCCCTTCTGCCTGA

CACCTTTCAAATTGATGACCAGCAGTACAACTATCAACAGTTTCTCTACAACAACTCTATACTACTGGAA

CATGGACTTACCCAGTTTGTTGAATCATTCACCAGGCAAATTGCTGGCAGGGTCGCCGGTGGCAGGAACG

TTCCGCCTGCGGTGCAGAAAGTAGCAAAGGCCTCCATTGACCAGAGCAGGCAGATGAAATACCAGTCTCT

TAACGAGTACCGCAAACGCTTTTTGCTGAAGCCCTATGAATCGTTCGAGGAACTTACAGGGGAGAAGGAG

ATGGCTGCCGAGCTGGAAGCGCTCTACGGCGACATTGATGCCGTGGAACTGTATCCTGCCCTGCTGGTGG

AGAGACCGCGCCCGGATGCCATCTTTGGGGAGTCCATGGTAGAAATGGGAGCACCGTTCTCCTTAAAAGG

ACTGATGGGTAATCCTATATGTTCTCCTAACTACTGGAAGCCAAGCACGTTTGGTGGAGAAGTCGGTTTC

AAAATCGTTAACACTGCCTCGATTCAGTCCCTCATCTGCAACAATGTGAAGGGCTGTCCCTTCACTTCAT

TCAACGTCCCAGATCCTCAGCTCACCAAAACAGTCACCATCAACGCGAGCGCCTCCCACTCCCGGCTAGA

GGACATCAATCCCACAGTCCTGCTGAAAGGCCGCTCCACTGAACTGTAGACGTCTGTTGATCGTATTTAT
TTATTTATGAGCCAGTTCTTTTAACTTAATTATTTAATAATATTTATAGTAAACTCCTGATGTTACCTGA
CATCTTCTGTAACAGAAGGCAGTACTCTTCTTGCAGAGAAGAGCTCATACTTGTGAAAACTTCCATGTCA
CTACTTTAAAGACATATTTTCTTCAAGTTTTCATTCTTTTTTATAAACCAATGGTAAATGGATTTTGACA
TCTTTTTACTTGAATTTAAATTCCTATGATGTTAGAAGAACAAAGTCAAAGATGTCTGAATGCTTAATGT
CACTGCAACATGGAAAACACTGCCGGTTTCTGTACTGTCAGATCTGTGTTTCTTCCATGATGCACTAGGA
GCAAAGAACATCTGGATTTTAAAGAACTACAGGGCATTTTTCTCTTTCATCAAGTAAAAAGAGATTCAAG
TCTGTTTTTAAAGGAATGTTTAGCTCAGACATTATCAATAATTTCATGTCTGCTTTTTTAAAGCAGCATT
GAAACAGTAATTTGAAATTTCTAAATTCACTGGGTAGAATCACTTTTAAAAATTTATTTTATTTTCTGTA
CCAAAGTTATAAACTTGTATTCACATCAATAGAGGAAACTTTCATAGATTTAAATCTCTAAAATCAGTGG
AAATTATACTACAAACTGCTGGTTAAAAGGTTGCCTAAGAGACTTTGCATTTTCACTAAGAGTAAAAATC
TAATGTGACTGTTACAACTTCCTTTCAAATAAAAATGCCAAATGTAGTAAGGTGGTGGAGCCACTGAGGT
CCTATCTTAAAATATTTTTTGTAGAGAGATTCCACAATTTGTTTTTGTGCCTAGTAACAAAACCTATGAT
AGCAATTTCTACATTTACAATCAGCAATAAAAATAGAAGAAAACCAAAACAGTATAAATTTGGGTTTAAA
CTCTTTAAGGAGACTTTACTTTAGCCTTGTGTGTACTACAGGCCTGGTACTCAACAGACTTCACTCTGAG
GTTCATGAACTTCCAAGCTGGCCTCACTGATGGGCTGTTTTCTTAGACTTTCTGTTGTACAGTGTGACTT
CGCAGTGGCCTCAGGAAATGCCTCTTCAGAATCCACTAGGCATTACTGAATCCATTCAGGCATTGCTTTC
ACCCAAATCTTGATCAGCCAGTTCAGTTGTGCATCGGAACCAAGTCGGGCCACACACATGCTGCTCCTCT
TACTGTTTCTCTTACAGCCTTCTGTGCTAAGAGAATCTCTTGTCTAACTGATGTATTTCTTCTGGTTTGT
TTTCCTGGTTTGAAGATGAGAGTTCTTCTTTCTTTGAATTCGACCAATATTTTCTTACTTGAAATTTTGC
AGTTTTCAGGAAAACCTCAGCTCAGGACTACTATTTAGTTCCTCCCAAGAGGAATAAAAGAGAAAATATG
GCGCTTAAAAAAAAAAAAAAAAAA

Oryctolagus cuniculus interleukin 1 beta (IL1B), mRNA

NCBI Reference Sequence: NM_001082201.1

GenBank Graphics

>NM_001082201.1 Oryctolagus cuniculus interleukin 1 beta (IL1B), mRNA
ACAGATCTCTTCGAGGCAAGAGGCACAACAGATCGCTTTGGGATTCTCTCCAGCCACTCTTCATTGTTCA
AATCTCTAGGACAGCCATGGCAACAGTACCTGAGCTCACCAGTGAGATGATGGCTTACCACAGTGGCAAT
GAAAATGACCTGTTCTTTGAGGCCGATGGTCCCAATTACATGAAGAGCTGCTTCCAGGACCTGGACCTCT
GCTGTCCAGACGAGGGCATCCAGCTGCGCATCTCCTGCCAACCCTACAACAAGAGCTTCCGGCAGGTCTT
GTCAGTCGTTGTGGCTCTGGAGAAGCTGCGGCAGAAAGCAGTTCCCTGCCCACAGGCCTTCCAGGATGAC
GGCCTGAGAACTTTCTTTTCCTTAATCTTTGAAGAAGAACCCGTCCTCTGCAACACCTGGGATGACTACA
GTCTTGAGTGTGATGCAGTACGATCACTGCACTGCAGGCTCCAGGATGCACAACAGAAATCCTTGGTGTT
GTCTGGCACGTATGAGCTGAAAGCTCTCCACCTCAATGCAGAGAATCTGAACCAACAAGTGGTGTTCTCC
ATGAGTTTTGTGCAAGGAGAAGAAAGTAACGACAAGATACCTGTGGCCTTGGGCCTCAGGGGGAAGAATC
TGTACCTGTCCTGCGTGATGAAAGACGATAAACCTACCCTGCAGCTGGAGAGTGTAGACCCCAACCGTTA
CCCAAAGAAGAAGATGGAAAAACGATTTGTCTTCAACAAGATAGAAATCAAGGACAAGCTGGAATTTGAG
TCTGCCCAGTTCCCCAACTGGTACATCAGCACCTCTCAGACAGAGTACATGCCCGTCTTCCTGGGAAACA
ACAGTGGCGGCCAAGACCTAATTGACTTCAGCATGGAATTCGTGTCTTCCTAAAGCAAGCCTTACAAGGA
GGGTCCGCGGTGCTGAATGTGTTCACCCCTGCGGCTGGCAGACGGGAAACAGATTGTGACACCCCACTGC

CTTCCCTTGGGAAAATTAAGGAGAGCTCTTTCCCACCAGCTGGGAGCAGCAGCTCTCTCCTCCCAGGGCC
ACTCCTCCGACCCTTGCTGAGCCAGCCTCTCTTACCTCTGCCATTCAGGCAAGGCCAGCCTGACAGTTCA
AAGAACCTTGGTACCCAGCTTCTATCAGCAACCACTAAATTATCTATTTATGTATTTATTGATAGGGTGG
TCTATTTAATCCAAGGAGACCAGAGGCAGCAGCATCTTCAGTATTTATGGAATAAATTTAATCTGAACTT
GTATGCTGTCTACATCAAGTCCTTTCACTAGGGCCAGAAGTATATAAGCTTGGGCTGAGTTATTTAAATA
GGAATAATTATGGGTGAAGAAGTATGATCACACTGTTTCAATGGTTCTGAAATAAACTATACTGGAAAAA
GTC

Oryctolagus cuniculus interleukin 6 (IL6), mRNA

NCBI Reference Sequence: NM_001082064.2

GenBank Graphics

>NM_001082064.2 Oryctolagus cuniculus interleukin 6 (IL6), mRNA
CGAGCCCACCRGGAACGAACAGAGCTCCCTCTGGCCTCCAGGAGCCCGACTATGAACTCCTTCACAAGCG
CCCTCCGCCCAGGTCCCCTCGGCTGCTCGCTGGCGCTGCTCCTGGTGGTGGCTACCGCTTTCCCCACTTC
AGCACCCGTGAGGGAGGACTCCAACACCAAGGCCAGCCCCGACAAAACCCTCACGCCGCCAGGCAGAACC
ATCGAGAGCATCCGGAGCATCCTGGAGACCATCAAGGAGCTGAGGAAAGAGATGTGTGACCATGATGTCA
ACTGCATGAACAGAAAGGAGGCACTGGCGGAAGTCAATCTGCACCTTCCAAGGCTGATAGAAGAAGACGG
ATGCTTCCCGCCGGCGGTGAATAATGAGACCTGCCTGCTGAGAATCACTTCGGGGCTGATGGAGTTCCGG
ATGTATCTCGAGCACCTTCAGGCCAAGTTCAGGAGTGACGAAGAAAACACCAGGGTCAGCATGGTGCTGA
AGAACATCCAACACCTGATCAAGACCCTGAGGCCAAAGGTCAAGAATTTGAATGAAGAAGCCACCCTCAA
GCCAGCCGTTGCGGTCAGCCTGATGGAGAACCTGCAGCAGAAAAACCAGTGGCTGAAGACGACCACGATC
CACTTCATCCTGCGCGGCCTCACAAACTTCCTGGAGTTCACCCTGCGTGCCGTGGACCTGATGGAGTGCG
GGTGTCCTTGCTTGCGGAATTTCATGGGCTCTGCCTCCCACGGTCAGAACACACCATCCTGTCCATTGGA
CACATAACTTAACATTGTTTACGAAACTAGGAAGATGAGCGTTAGGACACTATTTTAATTATTTTTTTAA
CTTATTAGTATTTAAATGTGGGAAGCTGGGTTAATTTATATGCGATTGATATTTATAACTTATGAAATGC
CATTTGTGATATTATAACTGTTACAGTTGTGAACGAATAGTGCACAATGGGCAATTTGAGTATCCCCTGT
TACAGAGCCAAATCTTTTCTTGGAATGTGTAGGCTTACCTCAAATGAATTGCTAACTTACACTTTTCAAG
AAATGTTTAAAGTATATTTATATATTGTTTAAACTATTCTTATACCAATAAATTACATTTTTTTAAAAAA
AAAAAAAAAAAA

Oryctolagus cuniculus caspase 3 (CASP3), mRNA

NCBI Reference Sequence: NM_001082117.1

GenBank Graphics

>NM_001082117.1 Oryctolagus cuniculus caspase 3 (CASP3), mRNA
ATGGAGAACAACGAAACCTCCGTGGACGCAAAATCTATCAAAAATTTGGAAACACAGACCATACATGGAA
GCAAATCAATGGACTCTGGGAAATATCTGGACAATAGTTACAAAATGGATTATCCTGAAATGGGTTTATG
CATAATCATTAACAACAAGAACTTTCATAAAAACACTGGAATGTCGTCCCGGTCTGGTACAGATGTAAAT
GCAGCAAACCTCGGGGAAACATTCATGAACTTGAAATACGAAGTCAGGAATAAAAATGATCTTACACGTG
AAGAAATCATGGAACTCATGTACAATGTTTCTAAAGAAGATCATAGCAAAAGGAGCAGTTTTATTTGTGT
GATTCTAAGCCACGGTGATGAAGGAGTCATTTATGGAACAAATGGACCTATTGAACTGAAGAAATTAACA

AGTTTCTTCAGAGGAGATTACTGTAGAAGTCTAACTGGAAAACCTAAACTTTTCATTATTCAGGCTTGCC
GAGGCACAGAGCTGGACAGTGGCATCGAGACAGACAGTGGGGTTGACTATGACATGGCATGCCAGAAAAT
ACCGGTTGAAGCCGACTTCCTGTATGCATATTCCACAGCACCTGGTTACTATTCCTGGCGAAATTCGGAG
GAGGGGTCCTGGTTCATCCAGTCCCTGTGTGCCATGCTGAAAGAGTACGCTCACAAACTTGAATTCATGC
ACATTCTTACTCGTGTGAACCGGAAGGTAGCGACAGAGTTCGAGTCCTATTCCCTTGATGCCACTTTTCA
TGCAAAGAAACAGATTCCATGTATTGTGTCCATGCTCACAAAAGAACTGTATTTTTATCACTAAAGAAAT
G

PREDICTED: Oryctolagus cuniculus BCL2, apoptosis regulator (BCL2), mRNA

NCBI Reference Sequence: XM_008261439.2

GenBank Graphics

>XM_008261439.2 PREDICTED: Oryctolagus cuniculus BCL2, apoptosis regulator (BCL2), mRNA

AAAAAAAAAATAAAACCCTCCCCCGCTCCCCACCCTGGCCGCACCACACACGGCGCGGGCTCCTCGCGAG
GCCGGCGCGTCCTGCCTTCATTGATCCGCAGCTTTTCGGAAAACGCGTCGGCTGCTCTCGGAGTTGAATC
AGAAGAGGACCCCGCGCCGGCCGGGCCCCGTCGGCGCCCGGGGCAGAGTCCGCGCCGTGCCCGCGCGGCG
CGCGCGCCTGTCAGTGGCCGAGGGGAGCCCGGACGGAGCGCGCTCCGGGGCTCGGGGCCGGATTCCTGCG
GATTGACGTTTCTGAGGAGCAGAAACCCGGGGGCCAGCCTGGAGACCCCCCCGCCCCGGGGTGCCTACAC
CCGCGCCCGCCGCCTCCCGATTCATTCGGAAGTTTCAAAGCGGCTCTCACCCGGGAGCTCCGAAGATTGA
TGGGATCCTTGCCTTATGCGTTTGTTTTGGTTTTGCAGAGAAGGAAACTGGACAGAGGGTCATGCTGTCC
TTTAAAAACACAACATCACGTAGGGAAAAGACTGGCGTTCACCACGCTTATGAATGATAACGTGCCTCGT
GAAACAGAGATCCGAAAGGGATTTGAGTAAAAATCTCCTGCGCCTGCTGCCAACGGGGAAACACCAGAAG
CGAGGGCTCCGCGTGACTGAAGACACCCCCGCGCCCGAGGATGCGGCGCGCGCCCGGGAACCCCGCCGGC
TTCTGAGCGGGCCCGTGCGGCGGGACGGGGCCGGAGGCACCGCGGGCGCCCAAAGGGCAGCATGGCGCAC
GCCGGGCGAACAGGGTACGACAACCGGGAGATCGTGATGAAGTACATCCACTATAAGCTGTCCCAGAGGG
GCTACGAGTGGGACGCTGGGGACGCGGGCGCCGCCTCCGCGCCGGGCGTCTTCTCCTCCCAGCCCGCGCC
CGCTGCGCCCCGGGACCCGGCCGCCAGGACCTCGCCGCCGCCGCCGCCGGCCGCCGCGGGGCCCGCGCTC
AGCCCGGTGCCACCTGTGGTCCACCTGACCCTCCGCCAGGCGGGCGACGACTTCTCCCGGCGCTACCGCC
GCGACTTCGCGGAGATGTCCAGCCAGCTGCACCTGACGCCCTTTCACGCGAGGGGGCGCTTTGCCACGGT
GGTGGAGGAGCTCTTCAGGGATGGGGTGAACTGGGGGAGGATTGTGGCCTTCTTTGAGTTCGGTGGGGTC
ATGTGTGTGGAGAGCGTCAACCGGGAGATGTCGCCCCTGGTGGACAACATCGCCCTGTGGATGACTGAGT
ACCTGAACCGGCACCTGCACACCTGGATCCAGGATAATGGAGGCTGGGATGCCTTCGTGGAACTGTACGG
CCCCAGCGTGCGGCCTCTGTCAGACTTCTCCTGGGTGTCTCTGAAGACTTTGTTCAGCCTGGCCCTGATA
GGAGCTTGCATCACCCTCGGTGCCTACCTGGGCCACAAGTGAAGACACCAGGCCTGCCCCCCACAGATAT
GCAAACAGTTCACGAAAGCAGGAGACATGATATGCATTGTCAGTGCCGCACCATGCAACGCAGCTGGCTC
TGTGAAGCGAGAGCCAGCAAGCCCCACGCGTACACAAAACAGCTTTCAGGCAAAATGTCGAATCAGCTAT
TTACTGCCAAAGGGAAATACCATTTATTTTTTACACTTTGAAGAAAAAAAGATTTATTTATTTAAGACAG
TCCCATCCAAACTCCTGTCTTTGGAAACCTTCCCACGAGTGGCCCGAGCACTCCTTGTGCGGCTGCCCCC
GGTGCCCCACACCCGTGGGCGTAGATTTGCTGGCCGTGTTCTTGGCCGCACTGAGCGACCATCTGAAGAG
CAAGAAAATGGACCCAGGGCTTGACCCCGGCAAGCCAGCTCTCGGACCTGTCTAGGCTGGGAGGAAGGTG
TCTTTCCGACCTGCATCTACCTGCCCTGGGGCCTGGGACGTCAACTGGGAAGTCCTCATTTCCTTGGCCT

CAAGAACACACACTTGGCCCGTGACCCACATGGTCCAGACCCAGTTGCAGGAAAGGAAAACAAACAAAAA
ATACCTGGGAGCTTCAGACGGACGTGGCCCCCGCTGCCCTTCCCACGCCCAAGGACGGCTGCGGGAACAA
AATGCCCTTAAACCATAGGAAAGTATTTTTTTAAGCTACCGATTGTGCCGAGAAGCGTTTCAGCAATGTA
TACCATGTCATCCAGTACCTTAAGCCCGGATTGTGTATATTCGTATATTTTAGATACGCAGTGCCCGCCG
CCCAGTACTGGCTGTGTCTGAGCCGGAGACAGCATCCTCTGGAGTTCAAGGCAGGGGACGTTTCGGTGAC
TTCTTCAACACAGGAAGGCTGGCCCAGAGCGTCAGGCTGCCACAAGTGCCTGCATTCCGGAGACGGCGTC
TGTAGAAGCTGCCTTGTGTCCGGCTCTGGGGCCTGGCCCTGGAACTGAGCCTGGGCACCCGCAGGCTCCT
GGTGGGGATTTTTCAACAGAGCGGTGCGGTCCTCGAGTGTTTGGAAGCTGATGCAGCTCAGAATTCCACT
GTCAAGAAGGAGCAATAGTGGGACTTGGCCCTGGGCCTGTTGCGCTCGGGCCGGCAGGCAGGCCTGTTCT
CTGGTAGAGCGTGGAACCTGGGGCCACAGCTCCCCTAAAGACTTCGTCACTGTGGAGGCAAGGAAAGAGG
CCGCGTGCGTCTCCTGGCAGAGGGACGTAATGGAGACTGAGCAGCCGCCACCGAAACGGGGTGCGGCCCG
TGGTGGCCGAGGCACAGGTGCATTGGCTGCTCGGCCGTGCGCCTGTCTGTGCAGTTTAAAGCAAGGTTTT
CAATGACTTTGAGTAGGGTCATGAGCCCTAAAGGAAGTGTTGAAATGAGGTGTCATGGATTAATTGACCT
GTGTCTGTGGAATTAACATGTAAAACATTATCTTGTCATTGGAGGTTGTTTTTATTTGAAAACCTGATAG
AAGAAAAAAAAAAATAGTTCCAGGTGTGGAATGTGGGGATTATCTGTACATCCTGGGGCATTAAAAAGGAA
GAAAAAAAACCAGTGGTGGAAAACTGTGGAGAAGTAACAGAAAAAGTGAGGTCTCTGGCTAATAAACTAG
CAAATACTTTCGCAAGTTTAGAATCAGCTTTGAGACATTCATGGAATAACTTTGTGGCATTATTGCATTA
TATACCATTTATCTGTATTAACTTTGGAATGTACTATGTTCAATGTTTGATGCTGTGGTTGATATTTCGA
AAGCTGCTTTTTGAAAAAATACATACATCTCAGCATTTTCTGAAATAATTGTATTTAGTTATGGCCTGCA
CACTATTTGTTAGCAAAAACGATCATTTTTCCATTGGAGATGCTTGTCTCTCGTTCCTTCAGAAGCGTTT
CTGAAAGGACGGGTCAGCCCTGAGTCTCGGCTGCCTGAGGAAGCCTGGGTTTTGCTGGCCACTGGGAGGC
TCGGATTTCACCGAGTTGCATGCAGCTCCATGTCGACAGAACTGCTCCGGGCTGTGGATGCATCCCCTTG
TCCCTTTGCCCTCGTGCCTTGAAGGGTTCCTTGGCCCTGGGCAAGTCACCATTTTGCTCACATAATTTAG
GATTCCATGCATGTGTGGGTAAACCCGGGAGATTCATTTGGTTGCAAACCCAGTGGGGAAGTGACCCAAG
GATGGACATCTGGCTCTGACGGCTGCCCCCTTTAGGCGACCTGTTTTGATGCAGACCCACCCCGACCCCC
CCGAAGGAGGCTTTTCTGCCGCTGCCCTTCGGGGCCTCCTGAGTGCCGTGGGGCTGCCACTTCGCAGGGA
GGGCAAGAAACCCACGGTTTGGAGCCAGACCTCCCTGGCGGGCCTCAGGGAATGGGACGATCAGACCTTT
GAATGATTCTAATTTTTAAGCAAAATATTATGTTATGAAAGGTTTACATTGTCAAAGTGATGAATATGGA
ATATCAAGTCCTGTGCTGCTATCCTGCCAAGATCACGTTCATGGAGTCAGTGTGCCGGACACGCCGAGGC
GCGAGACCCTCCAAGCTGCTTCAAAAGTCGCCACGAAGAACACGGACAGTTTTCATAACAGAAAGCCTGC
TCTCATCTTTTATTGTTTTTGTTATTGTTGTTCAAATTGGGATTTGCAGAGTATCTGAAAAAAAATGTAC
ATATAAACAGAATGGTCCAGGGGGCTACCCTCCAGGCTGGTTTGTCCTCAATGCACCCAGCCACCTGGCG
TGTACAGTCCCGTGGCTGTGCCCACTCCACGGGGAGTCGATGTTGCAGTTTTCCTTGTTGTTAAAGACAT
ACTAGAAGCAATGAATGTGTATAAAAGCCTCAACTACTCATTTTTTTTCCTCTTTCCTTTCTTCATTGGC
TCAGTTAGTTGGCAACTGGGCAACAGAGAACCATCCTGAAGTGTTTCGTGTTGAGAAGGGATTCATACCC
AAATCTTAACACACAGAGAGGAGTCCTCCTTCTGTTGGCCCAGGTCCCAGTTAAAGAGGACACCTTCACT
TTGCAAATTGTGGTCAAGGGCTTTAAAAAAGAAACCAGGAGGCAAAGAGCATCCAAGAACCTCCCTGTTC
CTCCCAAGCCTCACCATCCCAGCACTCAAAGAAAAGACCCAAACAATGTAGAGAAGAGTTCATCCCAGCT
TTTGCAGATTTAAGATTTGGATTAAAGTCCCACTTAGAATTAAGGAAGCACTTTTCTGTAGTTCAAACGA
AACCCCACCATCTCTGCCCCAGCTCACTGTGTCTCTGTGCATTCACGTGTGCCCTGCCATTTACATGTGA
CTTTTATTTCATTAAGAAATGTTTGTTACCTATCACCTGGAGACCTAGCCCAGGCCCAGTATTGGGGACA

GCAGGAAGGGATGATATATCAGAAGGGTAAGTTGTAATAATCAGGACTAAATGTAAATCATTAGGGCAGT
CCCAACAAATGTCTAGCTTTCACATCCCGAATCTAGGGAATGAACAGAATTGCAGTAGTCTATATTTGTA
AAGCTATAGTAAAACAAATAATTTATAAATGTGAACTTAAACTAATTCCAATTGTCCTTTGAAGGCAATG
GCTGTTTTTATACTTTCTTATCACTTATAGTTAGTAACATACACCTCCTCTATCAGAAAAAAAACAGGAA
AGACTTGAAACATAAGCTGTTCTAAGGAAATCAGGGCTTCAGTTGAAATCCTATTCTGATCTTCCTCCGT
GGTGTCCTTTGCAGCCCAGACAAATGTGGTTGCACACTTTTTAAGAAATACAATTCTACATTGTCAGGCT
TATGAAGGTTCCAATCAGATCTTTATTGTTACTCAATTTGGATCTTTCAGGGATTTTGCTTTTTAATTTC
TACAAGACAAAGGACATTTGTTGTGGGTGGGAGGGGAGAAGAATTTTTAAATGAGTAAAATATTCCCAAG
ATTGGATCAGGGAATGGAGGTTTTCAGAGAAGCCAGATGTATGGGGGGGGGGGGGGGTGAGGGATCTTTA
GAAGGGTCCGTGTCATTCCTCCATAAAGGCCCTTATGCAACAGATGAGAAACATTTTTTTAAGTCCTGTG
GTGCTTTCAGATTTGGAGATACTGGCACACTCAAAGGCCAGCTCAGTGTGGCTGCGCAGGGGTATAAAGT
TTAAAGCTGGGTGTGTGTAATGGGAATGTTAGCACAAAGTTTGCAGTTGGAACTAACAGGATATTTAATG
ACAATGACAACTTTCTGTTGGTAGGAACATCTGTTTTAAAATATTTATTATGCAGGACACAGAAAACATT
TAAATAGAATGAAGTTGAATAAAACAATGGAAAGCTAAATTGGGTCCCTTAGCCTTACCCAACGACTCAT
TCTGTTCTGTGCCTTTGGACAACCATGACCTGGGACAACCATGAAATTGGGCACAAAGAAAACCAGATGG
AATATGGATGGTACTGCAACAGTTGCGTCAAGACGATCCCAGAAAAATAACTTAAAACCAAATTCCTGTT
GTTAATAATTTTTCTCTATGTACCAAGATCAGCACCCTCTGTTAAGCACTCATGGAAGATGGAATGTTAA
TAAATACGACCGTAGTGTTGACACTCCATAATGCCATGCACCTAAATATCTTGGAAAATCTGCTCTGCGG
CCTCCAGCTAGCTCATTTCATTAAGTTTTGCCCTCCAAGGTAGCATTTGAAGGAGTGATGGCTGATCAAA
TTTCCTTTGGGGAAGCTTCCTTCTGGTGGTTGTGTTTGTTATTTTCTTTAAACTAATTTTTGCTTTTGTT
TTTGAGTTACTGGGGTTATTTTCATTTTAAATAAAGTAACTGTACAATATGTATTTTTGTATTGAAAGCT
TTTGTTGTCAAGATTTTCATACTTTTACCTTCCATGGCTGTTTCTAAGATTGATAATTCTTAGCAGTGGC
TCATAGCCTGAAATACTGTATACACCAAAAATGTATGGTAAGCATACTTTACATAGTTAAGGTAAAGTAA
GTTTCCAGTTGGCCACCATTAAATATAATGGCACTTTGTGTTGTTGGGAAAAGTCACATTGCAATTAACC
TCTCCTTGTCTGTCCAGTTAATTTCGTGAAGAAAAATAAAGTACAGTGTGAGATA

PREDICTED: Oryctolagus cuniculus BCL2 associated X, apoptosis regulator (BAX), transcript variant X1, mRNA

NCBI Reference Sequence: XM_008252361.2

GenBank Graphics

>XM_008252361.2 PREDICTED: Oryctolagus cuniculus BCL2 associated X, apoptosis regulator (BAX), transcript variant X1, mRNA
AGGGCGAGTCCCGTGGCCGGCCAGCCCCCGGATCCCTCCTGCCGGCCCGGGGCCGTGCGATCTCCAAGCA
CTCCGGGCCAGGAACTCCCGGATCGGGCGCTGCCAGCCTCCAGTCCCCTCCCTCCTTGGAGGTTCCCGGC
TCTCCGGCCTCCACGCCCCGATCCCTTCCTGCCTGGCGTTCTCGGACCCCTGATAACTTGGGGATCCCGG
AACCCCAACTCCCGGGCAGGCCCGGGCTTGTCGCGGGCACCACTTCCTGCCTCTGACCCCGGTGGGAGGG
GCGGTCCTGCCTCGGCCCCCTCAGACTAGGGGTCTGGGTGCAGGAGACGCGCCCTGAGCTCCCCTCCCGG
AAGAATGTAGGATGCGGGCCCCGCTGCCAGCCCCTGGCTCCTGCAGAGCCTCACTGCATGGACTCGCAGC
CGCCTTGTGCACCAGGGACCCCGGAGTTCGCGGCACGGCCCTCCCTCGGGGACCACGGGCCTCCCAGGAG
TCCAGGCGCCTCTTCCCTCCTCTCTCCTCTAGGGCCCACCAGCTCTGAGCAGATCATGAAGACAGGGGCC
CTTTTGCTTCAGGGTTTCATCCAGGACCGGGCCAGCCGGATGGGGGGGCAGACGCCCGAGCTGGCTGTGG

CGCAGGGGCCCCAGGACGAGTCCACCAAGAAGCTGAGCGAGTGTCTCAAGCGCATTGGCGACGAACTGGA
CAGTAACATGGAGCTACAGAGGATGATCGCTGCCGTGGACACGGACTCCCCCCGCGAGGTCTTTTTCCGA
GTGGCAGCCGACATGTTTGCCGACGGCAACTTCAACTGGGGCCGCGTTGTCGCCCTGTTTTACTTTGCCA
CCAAGCTGGTACTCAAGGCCCTGTGCACCAAGGTGCCTGAGCTCATCAGGACTATTATGGGCTGGACGCT
GGACTTCCTCCGAGAGCGGCTGCTGGGCTGGATCCAAGACCAGGGTGGTTGGGACGGCCTCCTCTCCTAC
TTCGGGACCCCCACGTGGCAAACGCTGACCATCTTGGGGGCCGGAGTGCTCACCGCCTCACTCACCATCT
GGAAGAAGATGGGCTGAGGCCGCCAGCTGCCTTTGGACTGTGCCATTTCCGCATAAATTATGACATTTTT
CTGGAAGGCTGGGGATCCGGGGACACAGGCGTTTTTCTTACTTTTGTAATTATTGGGGGTGGGGAGGGGA
GGGAGGCCTGGGCGGGGGCAATAAACCTCCTTTGGGCCGCA

Oryctolagus cuniculus glyceraldehyde-3-phosphate dehydrogenase (GAPDH), mRNA

NCBI Reference Sequence: NM_001082253.1

GenBank Graphics

>NM_001082253.1 Oryctolagus cuniculus glyceraldehyde-3-phosphate dehydrogenase (GAPDH), mRNA
CGCTCCCGTTGCTGTCGCCCGTTCGACAGGCAGCCGCTTCTTCTCGTGCAGTGCTAGCGCGTCCCCGAGA
CACGATGGTGAAGGTCGGAGTGAACGGATTTGGCCGCATTGGGCGCCTGGTCACCAGGGCTGCTTTTAAC
TCTGGCAAAGTGGATGTTGTCGCCATCAATGATCCATTCATTGACCTCCACTACATGGTCTACATGTTCC
AGTATGATTCCACCCACGGCAAGTTCCACGGCACGGTCAAGGCTGAGAACGGGAAGCTGGTCATCAACGG
GAAGGCCATCACCATCTTCCAGGAGCGAGATCCCGCCAACATCAAGTGGGGTGATGCTGGTGCCGAGTAC
GTGGTGGAATCCACTGGCGTCTTCACCACCATGGAGAAGGCCGGGGCTCATTTGAAGGGCGGAGCCAAAA
GGGTCATCATCTCAGCCCCCTCCKCCGATGCCCCCATGTTTGTGATGGGCGTGAACCACGAGAAGTATGA
CAACTCTCTCAAGATTGTCAGCAACGCATCCTGCACCACCAACTGCTTAGCCCCCCTGGCCAAGGTCATC
CACGACCACTTCGGCATTGTGGAGGGGCTCATGACCACGGTGCACGCCATCACTGCCACCCAGAAGACCG
TGGACGGGCCCTCTGGGAAGCTGTGGCGTGACGGCCGCGGGGCCGCCCAGAACATCATCCCTGCCTCCAC
TGGCGCTGCCAAGGCCGTGGGCAAGGTCATCCCCGAGCTGAACGGGAAACTCACTGGCATGGCCTTCCGT
GTGCCCACCCCCAACGTGTCGGTCGTGGACCTGACCTGCCGCCTGGAGAAAGCTGCTAAGTATGACGACA
TCAAGAAGGTGGTGAAGCAGGCATCCGAGGGCCCCCTGAAGGGCATCCTGGGCTACACCGAGGACCAGGT
CGTCTCCTGCGACTTCAACAGTGCCACCCACTCCTCTACCTTCGATGCTGGGGCTGGCATTGCCCTCAAT
GACCACTTTGTGAAGCTCATTTCCTGGTATGACAACGAATTTGGCTACAGCAACAGGGTGGTGGACCTCA
TGGTCCACATGGCCTCCAAGGAGTAAGAGCCCTCAAACCACCGGCCCCAGCGAGAGCACCAGAGGAGGAC
GAGAGGCCCTCAGCCGCTGGGGAGTCCCTGCCCCCACTCAGTCCCACCACGCTGAGAATCTGCCCCTCTT
CACAGTTTCCATCCCAGACCCCCTGGAGGAGGGGAGGGGCTTAGGGAGCCCTACCTTGTCATGAAACCAT
CAATAAAGTCTCGACAGCAACG
